# Phase I Study of Ficlatuzumab and Cetuximab in Cetuximab-Resistant, Recurrent/Metastatic Head and Neck Cancer

**DOI:** 10.3390/cancers12061537

**Published:** 2020-06-11

**Authors:** Julie E. Bauman, James Ohr, William E. Gooding, Robert L. Ferris, Umamaheswar Duvvuri, Seungwon Kim, Jonas T. Johnson, Adam C. Soloff, Gerald Wallweber, John Winslow, Autumn Gaither-Davis, Jennifer R. Grandis, Laura P. Stabile

**Affiliations:** 1 Division of Hematology/Oncology, Department of Medicine, University of Arizona, Tucson, AZ 85724, USA; 2 Department of Medicine, University of Pittsburgh Medical Center, Pittsburgh, PA 15213, USA; ohrj@upmc.edu (J.O.); gaitherdavisa@upmc.edu (A.G.-D.); 3 Biostatistics Facility, UPMC Hillman Cancer Center, Pittsburgh, PA 15213, USA; weg@pitt.edu; 4 Division of Head and Neck Surgery, Department of Otolaryngology, University of Pittsburgh, Pittsburgh, PA 15213, USA; ferrrl@upmc.edu (R.L.F.); duvvuriu@upmc.edu (U.D.); kimsw2@upmc.edu (S.K.); johnsonjt@upmc.edu (J.T.J.); 5 UPMC Hillman Cancer Center, Pittsburgh, PA 15213, USA; soloffa@upmc.edu; 6 Department of Cardiothoracic Surgery, University of Pittsburgh, Pittsburgh, PA 15213, USA; 7 Monogram Biosciences Inc., South San Francisco, CA 94080, USA; Wallweg@labcorp.com (G.W.); Winslj2@labcorp.com (J.W.); 8 Department of Otolaryngology-Head and Neck Surgery, University of California-San Francisco, San Francisco, CA 94115, USA; Jennifer.Grandis@ucsf.edu; 9 Department of Pharmacology & Chemical Biology, University of Pittsburgh, Pittsburgh, PA 15260, USA

**Keywords:** HNSCC, cetuximab, ficlatuzumab, EGFR, HGF, cMet

## Abstract

Cetuximab, an anti-EGFR monoclonal antibody (mAb), is approved for advanced head and neck squamous cell carcinoma (HNSCC) but benefits a minority. An established tumor-intrinsic resistance mechanism is cross-talk between the EGFR and hepatocyte growth factor (HGF)/cMet pathways. Dual pathway inhibition may overcome cetuximab resistance. This Phase I study evaluated the combination of cetuximab and ficlatuzumab, an anti-HGF mAb, in patients with recurrent/metastatic HNSCC. The primary objective was to establish the recommended Phase II dose (RP2D). Secondary objectives included overall response rate (ORR), progression-free survival (PFS), and overall survival (OS). Mechanistic tumor-intrinsic and immune biomarkers were explored. Thirteen patients enrolled with no dose-limiting toxicities observed at any dose tier. Three evaluable patients were treated at Tier 1 and nine at Tier 2, which was determined to be the RP2D (cetuximab 500 mg/m^2^ and ficlatuzumab 20 mg/kg every 2 weeks). Median PFS and OS were 5.4 (90% CI = 1.9–11.4) and 8.9 (90% CI = 2.7–15.2) months, respectively, with a confirmed ORR of 2 of 12 (17%; 90% CI = 6–40%). High circulating soluble cMet levels correlated with poor survival. An increase in peripheral T cells, particularly the CD8^+^ subset, was associated with treatment response whereas progression was associated with expansion of a distinct myeloid population. This well-tolerated combination demonstrated promising activity in cetuximab-resistant, advanced HNSCC.

## 1. Introduction

Head and neck squamous cell carcinoma (HNSCC) is a morbid and lethal malignancy associated with chronic tobacco exposure or oropharyngeal infection with oncogenic human papillomavirus (HPV) [1]. Despite increased understanding of the genetic and viral underpinnings of HNSCC, 5-year overall survival (OS) in high risk disease remains 40–60%. Recurrence or metastasis following curative-intent therapy represents the major cause of death. Options for palliative management include the cytotoxic chemotherapies platinum, 5-fluorouracil, and taxanes; the anti-epidermal growth factor receptor (EGFR) human-murine IgG1 monoclonal antibody (mAb) cetuximab; and the immune checkpoint inhibitors targeting the programmed death-1 (PD1) receptor, pembrolizumab and nivolumab [2,3,4]. Currently, there is no standard therapy for pan-refractory patients, who will succumb with a median OS of less than 6 months.

Ubiquitous tumor overexpression of the EGFR receptor tyrosine kinase (RTK) compelled the development of EGFR inhibitors for the treatment of HNSCC [5,6]. Despite aberrant EGFR signaling in the majority of HNSCC cases, the modest clinical activity of cetuximab has been disappointing and de novo or acquired resistance is inevitable [7]. Unlike colorectal cancer, where activating *KRAS* and *BRAF* mutations predict cetuximab resistance [8], no predictive biomarker has been identified in HNSCC [9,10]. A likely resistance mechanism to anti-EGFR therapy is compensatory activation of alternate RTKs. The *MET* oncogene encodes cMet, an RTK bound exclusively by the ligand, hepatocyte growth factor (HGF). Overexpression of cMet transforms normal epithelial cells and enhances motility, invasion, and metastasis [11]. cMet and/or HGF are overexpressed in approximately 80% of HNSCC [12]. cMet activation is an established driver of epithelial-to-mesenchymal transition, a phenotype associated with cetuximab resistance in HNSCC [13,14].

Several lines of evidence developed in our laboratories indicate that cMet plays an important role in tumor-intrinsic resistance to EGFR inhibition. In vitro, the EGFR ligand transforming growth factor α (TGFα) stimulated activation of cMet in HNSCC cell lines. Dual inhibition of EGFR and cMet maximally inhibited phosphorylation of MAPK and Akt compared to single inhibition of either RTK, abrogating cross-talk. In vivo, dual inhibition retarded tumor growth, decreased the proliferative index, and enhanced apoptosis compared to either single agent [15]. Others found that dual blockade of cMet and EGFR was synergistic in erlotinib-sensitive HNSCC cell lines [16]. Growth factors have the potential to drive resistance to tyrosine kinase inhibitors (TKIs); in kinase-addicted cell lines, HGF rescued cells dependent upon HER2 amplification, NRG1 autocrine stimulation, *EGFR* mutation, and *BRAF* mutation [17]. In *EGFR* mutant lung cancer, cMet amplification and increased tumoral HGF expression are common mechanisms of both de novo and acquired resistance to EGFR TKIs [18,19]. Finally, serum levels of HGF have been associated with resistance to EGFR inhibitors in *KRAS* wild-type metastatic colorectal cancer and lung cancer [20,21,22].

A second important mechanism of action of cetuximab is antibody-dependent, cell-mediated cytotoxicity, triggered by engagement of its IgG1 Fc with the Fc receptor (FcR) on natural killer (NK) cells [23,24]. Mechanistically, cetuximab-activated NK cells upregulated human leukocyte antigen-C (HLA-C) on HNSCC cells via interferon gamma (IFNγ) [25]. Clinically, HNSCCs that responded to cetuximab were shown to have an increased rate of HLA-C mutations compared to non-responders or untreated tumors, which may contribute to immune evasion in the setting of cetuximab treatment [25]. Recent studies show that HGF/cMet signaling also orchestrates immune responses. However, this is not sufficiently understood [26]. Some studies identify HGF as a negative regulator of dendritic cell (DC) function and T lymphocytes [27], while others imply an immunostimulatory role by promoting recruitment of DC, B cells and T lymphocytes [28]. Thus, an immunological mechanism may exist for HGF/cMet-directed agents as well.

Because the HGF/cMet signaling pathway converges with the EGFR network at multiple downstream nodes, we hypothesize that HGF/cMet pathway inhibition may overcome clinical cetuximab resistance. We conducted a Phase I study evaluating the combination of ficlatuzumab (AV-299), a humanized anti-HGF IgG1 mAb, and cetuximab in patients with recurrent/metastatic HNSCC. We sought the recommended Phase II dose (RP2D) for subsequent randomized evaluation and explored mechanistic proteomic, signaling and immune biomarkers that may be associated with clinical benefit.

## 2. Results

### 2.1. Patient Characteristics

Thirteen patients were enrolled and received at least one dose of protocol treatment between September 2015 and June 2016. Baseline demographic and disease characteristics are summarized in Table 1, and are typical of a pan-refractory HNSCC population. The majority of subjects were male, median age was 58.4 years, and 12 of 13 (92%) had HPV-negative disease. The majority of subjects (92%) met protocol-specified criteria for platinum and cetuximab resistance, and 9 of 13 (67%) were VeriStrat poor. Although the trial was conducted prior to the U.S. FDA approvals for the anti-PD1 mAb, pembrolizumab and nivolumab, five (38%) had received prior anti-PD1 or PDL1 mAb in the setting of a clinical trial. In the 12 cetuximab-resistant patients, the median time from most recent cetuximab exposure was 17 weeks (range 2–44 weeks).

### 2.2. Dose-Limiting Toxicities and Recommended Phase II Dose

Adverse events are described regardless of attribution in Table 2, and reflect the expected, additive patterns observed during studies of ficlatuzumab or cetuximab monotherapy. The HGF/cMet inhibitor class toxicity of edema was observed in three subjects (25%), with one instance of Grade 3 peripheral edema. Due to the unique risk for head and neck edema in this patient population [29], this toxicity was specifically assessed at each study visit and was observed in three patients (23%), none requiring dose reduction of ficlatuzumab. No DLTs were observed at any dose tier, thus only Tier 1 and Tier 2 were utilized. Three subjects were treated at dose Tier 1, as the second subject had not cleared the DLT observation period when the third patient was enrolled thus criteria for escalation had not yet been met. Ten subjects were subsequently treated at the Tier 2 dose of ficlatuzumab 20 mg/kg and cetuximab 500 mg/m^2^ every 2 weeks. One subject (patient 6) withdrew consent during the DLT observation period following an unrelated thromboembolic event and was considered unevaluable for both DLT and disease response, but was included in Table 2. When 6 subjects on Tier 2 had completed the DLT observation period without DLT, Tier 2 was declared the RP2D. Three additional subjects were then enrolled to Tier 2 in order to reach the target of 12 biomarker-evaluable subjects.

### 2.3. Preliminary Oncologic Efficacy

**Figure 1:**
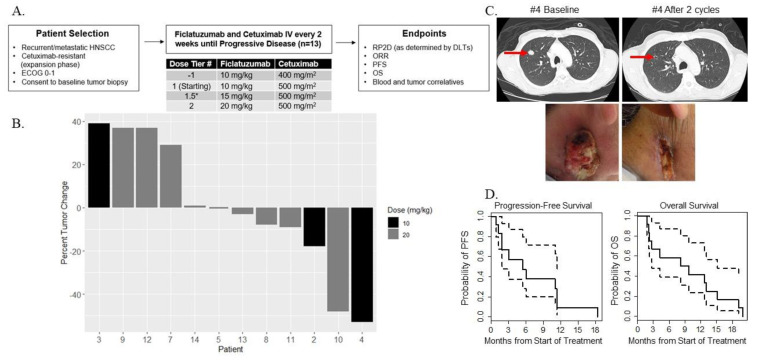
(**A**) Dose tier escalation rules and treatment schema. (**B**) Waterfall plot showing percent change in tumor burden by patient and by ficlatuzumab dose. Twelve patients were treated—three at 10 mg/kg (black bars) and nine at 20 mg/kg (gray bars) ficlatuzumab. (**C**) Representative lung CT images (top) and clinical photographs (bottom) of patient #4 at baseline and after two treatment cycles. (**D**) Kaplan–Meir curves (solid lines) with 95% CIs (dotted lines) showing PFS (left panel) and OS (right panel) in months. Time to progression and death were measured from first day of on-protocol treatment.

The protocol treatment schema is outlined in Figure 1A and described in the Treatment Plan in Materials and Methods section. The median number of cycles received was 3 (range 1–20). Twelve subjects were evaluable for efficacy parameters. There was no dose–response relationship between ficlatuzumab and percent tumor change according to RECIST, thus secondary analyses were pooled across the two dose tiers. The ORR was 2 of 12 patients (17%; 90% CI = 6–40%), with 1 confirmed partial response (PR) observed at each dose tier as depicted in the waterfall plot (Figure 1B). The partial response of patient #4 is illustrated by CT scans and clinical photographs, obtained at baseline and after two treatment cycles (Figure 1C). An index metastatic lesion in the right lung regressed to form a thin-rimmed cavitary lesion, while the exophytic, ulcerative right neck mass grossly flattened and epithelialized. The median PFS was 5.4 months (90% CI 1.9–11.4 months) and median OS was 8.9 months (90% CI 2.7–15.2 months) (Figure 1D).

### 2.4. Prognostic Biomarkers

We examined baseline plasma levels of HGF and scMet as well as IL6, a cytokine known to be regulated by HGF [30], for correlation with outcomes among the 12 evaluable study participants. All patients had detectable levels of HGF and scMet in baseline plasma. Two of 12 subjects (17%) had undetectable IL6 at baseline. None of the circulating markers were associated with tumor change. Baseline levels of scMet were inversely associated with PFS (HR = 1.92; *p* = 0.048) with a similar, but non-significant association observed for OS (HR = 1.63; *p* = 0.113); no association was observed between baseline HGF or IL6 and PFS or OS (Table 3). At the end of cycle 1, HGF levels increased from a median baseline of 0.9 ng/ml to a median of 17.9 ng/ml and continued to show a non-linear increase that eventually plateaued or decreased over time (Figure S1). In three patients whose blood was available at time of disease progression, HGF levels declined at time of progression. The increase in circulating HGF is consistent with a pharmacodynamic effect of HGF mAb treatment, as demonstrated in previous clinical studies [31,32,33]. scMet and IL6 levels did not change appreciably after treatment. No correlations among baseline circulating markers were found. The soluble cMet protein has been shown to correlate with tumor cMet protein expression in patients with non-small-cell lung cancer (NSCLC) [34]. Here, soluble HGF was correlated with tumor cMet (r = 0.62, *p* = 0.056), HGF (r = 0.54, *p* = 0.106) and the cMet–HGF complex (r = 0.65, *p* = 0.044) and soluble cMet was correlated with the cMet–HGF complex (r = 0.58, *p* = 0.0677).

**Figure 2:**
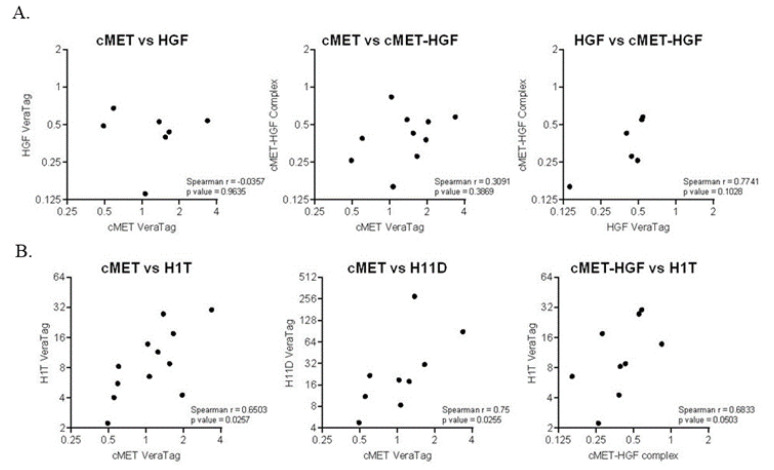
(**A**) Correlations between tumor cMet, HGF and cMet–HGF complex measured by VeraTag assays. (**B**) Correlations between cMet, EGFR (H1T) and EGFR homodimers (H11D) and the cMet–HGF complex and EGFR (H1T). Spearman correlation coefficients and *p* values are shown.

We next measured the cMet–HGF complex as a measure of cMet activation as well as total cMet and total HGF using the VeraTag assay in FFPE tumor tissue. None of the tumor markers were associated with change in tumor size. Neither total cMet nor HGF significantly correlated with the cMet–HGF complex (Figure 2A). While there was a non-significant trend towards poor survival, high total tumor HGF was not an indicator of OS (HR 1.47, *p* = 0.618) nor PFS (HR = 1.65; *p* = 0.508) (Table 3). Similarly, high activated cMet was not associated with OS (HR = 1.84; *p* = 0.390) or PFS (HR = 2.11; *p* = 0.273). We also measured total EGFR (H1T) and EGFR homodimers (H11D). High total EGFR, also trending towards worse survival, was not associated with PFS or OS when considering the adjusted *p* value, nor was the EGFR homodimer (Table 3). Furthermore, we observed a significant correlation between total cMet and total EGFR (*p* = 0.03) and the EGFR homodimer (*p* = 0.03), as well as between activated cMet and total EGFR (Figure 2B), suggesting cross-talk between these two pathways in cetuximab-resistant HNSCC.

Taking the lead from the Phase II study of gefitinib with or without ficlatuzumab in NSCLC, demonstrating benefit from ficlatuzumab in the VeriStrat poor subgroup, we also assessed serum VeriStrat, a proteomic classifier, in our study population [35]. Eight RECIST-evaluable subjects were classified as VeriStrat poor, while 4 were classified as VeriStrat good. There was no association between VeriStrat status and PFS or OS.

### 2.5. Immune Correlatives

**Figure 3:**
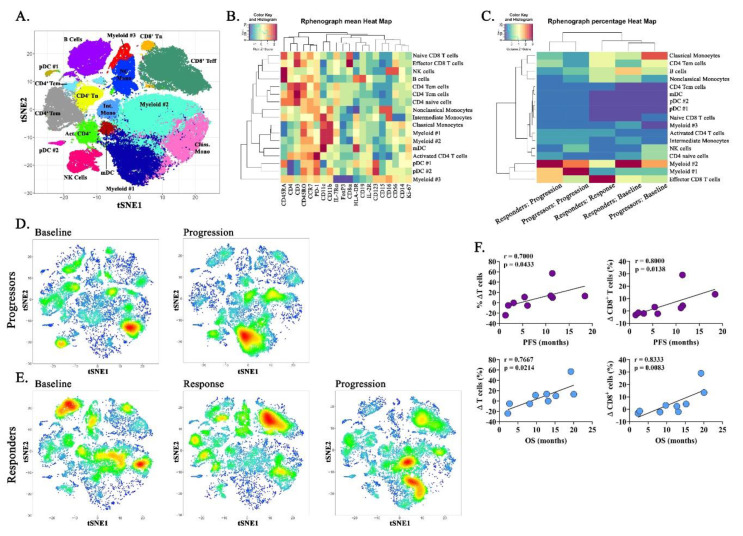
Immunophenotyping of peripheral blood mononucleocytes (PBMCs) identifies unique immune profiles in PD and PR subjects. (**A**) PBMCs from responders (*n* = 2) or rapid progressors (*n* = 2) were assessed by spectral cytometry (21 color) and analyzed using Rphenograph in Cytofkit for unbiased population discovery. Combined analysis of responding and progressing patient subsets at baseline, at response, and at progression illustrated as a t-SNE plot. (**B**) Heatmap depicting antigen expression of selected phenotypic markers corresponding to cell subsets in Rphenograph t-SNE (**A**). (**C**) Heatmap illustrating the proportion of cell subsets expressed within responders or progressors (*n* = 2 per group per timepoint) during baseline, response (after two treatment cycles), and progression. t-SNE density plots illustrating the increased proportion of cell subsets corresponding to Rphenograph for (**D**) rapid progressors and (**E**) responders. (**F**) Spearman correlations among all evaluable subjects between the change in percentage of total CD3^+^ T cells or CD3^+^CD8^+^ T cells with progression-free survival or overall survival, as indicated.

To investigate alterations in systemic immune composition during therapy, we employed polychromatic spectral cytometry to simultaneously identify major lymphoid and myeloid cell subtypes present within peripheral blood mononucleocytes (PBMCs). Of the 12 evaluable study subjects, 92% (11/12) were evaluated at baseline, 83% (10/12) were evaluated at progression, and two patients who exhibited a PR to therapy were examined during treatment response (17%; 2/12). To facilitate population discovery, unbiased computational analysis was conducted using Rphenograph in Cytofkit, a graph-based partitioning method which dissects nearest-neighbor data into phenotypically coherent populations based on relatedness. FACS data from the two subjects with the greatest tumor volume increase during progressive disease (“rapid progressors,” patients 3 and 9) and the two subjects with the largest reduction in tumor volume during partial response (“responders,” patients 4 and 10) were compiled at three time points: at baseline, at response (if responder), and at progression. These data were used to generate t-SNE plots and associated heatmap for data reduction and illustration (Figure 3A,B). At baseline, rapid progressors were characterized by high levels of classical monocytes and an undefined subpopulation of myeloid cells (myeloid #2) (Figure 3C,D). Upon progression, PBMCs from rapid progressors consisted predominantly of two myeloid subsets, both the previously observed myeloid #2 cells and the emergence of a unique myeloid #1 population. Both myeloid populations were characterized by high CD11b and CD11c expression and low expression of MHC class II (Figure 3B). Like subjects with progressive disease, responders were enriched in the myeloid #2 subset at baseline, yet these subjects also possessed an elevated proportion of peripheral B cells and a minor population of effector CD8^+^ T cells (Figure 3C,E). Notably, during response to treatment, responders demonstrated robust expansion of effector CD8^+^ T cells (Figure 3C,E). When subpopulations were analyzed via conventional gating strategies, this represented an 83.3% increase in CD3^+^CD8^+^ T cells during treatment response. Interestingly, upon disease progression in previously responding patients, subjects displayed an increased proportion of the unique myeloid #1 subset previously observed during disease progression in rapid progressors, as well as the myeloid #2 population conserved throughout all three time points (Figure 3C,E). Analysis of the change in immune cell proportions from baseline to progression identified a significant positive correlation between the increase in percentage of total CD3^+^ T cells and CD3^+^CD8^+^ T cells with increased PFS and OS (Figure 3F).

## 3. Discussion

Despite aberrant EGFR signaling in the majority of HNSCC cases, the modest clinical activity of cetuximab has been disappointing and either primary or acquired resistance is inevitable. Currently, there is no standard therapy for patients with recurrent/metastatic HNSCC after failure of platinum, cetuximab, and anti-PD1 mAb, and all such patients will succumb with a median survival of less than 6 months. The lack of therapeutic options represents a major unmet clinical need. Co-targeting EGFR and a parallel or compensatory oncogenic pathway may overcome cetuximab resistance. Dysregulation of the HGF/cMet pathway is one of the major mechanisms of resistance to EGFR-targeted therapy. In this Phase 1 study, we investigated the combination of cetuximab with the anti-HGF mAb, ficlatuzumab, in patients with cetuximab-resistant recurrent/metastatic HNSCC. The combination proved to be tolerable, with no DLTs observed at any dose level, thus the escalation–de-escalation rules were not triggered and the RP2D was identified efficiently. The RP2D is cetuximab 500 mg/m^2^ and ficlatuzumab 20 mg/kg every 2 weeks, which represents the full therapeutic dose of cetuximab and the maximal tolerated dose of ficlatuzumab monotherapy. Preliminary oncologic efficacy in this heavily pre-treated population was encouraging, with a confirmed response rate of 17% (90% CI = 6–40%) and a median PFS of 5.4 months (90% CI = 1.9–11.4 months), warranting formal Phase II study.

Ficlatuzumab has been combined safely with full-dose anti-EGFR TKIs and evaluated in patients with advanced NSCLC. A Phase 1b trial of ficlatuzumab plus the EGFR TKI gefitinib in Asian patients with advanced NSCLC demonstrated acceptable tolerability with an objective response rate of 33% [33]. However, a randomized Phase II study comparing gefitinib with or without ficlatuzumab failed to confirm clinical benefit from the combination [35]. In the subgroup of patients classified as VeriStrat poor and *EGFR* mutant, the addition of ficlatuzumab to gefitinib improved both PFS (7.4 months vs. 2.3 months; *p* = 0.02) and OS (23.9 months vs. 5.8 months; *p* = 0.04). However, further prospective study has been precluded by the rarity of this subpopulation [35]. The Phase I combination of ficlatuzumab with full-dose erlotinib also identified 20 mg/kg ficlatuzumab as the RP2D, with preliminary anti-tumor activity and manageable adverse events [36]. Rilotumumab, an IgG2 anti-HGF mAb, has also been evaluated in combination with the EGFR TKI erlotinib, demonstrating clinical efficacy in certain NSCLC subpopulations [31]. However, further development of rilotumumab has been halted due to THE lack of efficacy in gastric cancer trials. Small-molecule cMet TKIs are currently being evaluated in combination with EGFR TKIs in patients with EGFR TKI-resistant and cMet-amplified NSCLC, based on promising antitumor activity found in preclinical models [37]. 

In order to inform Phase II development of this combination in recurrent/metastatic HNSCC, we evaluated mechanistically relevant, tumor-intrinsic biomarkers that may point towards a subgroup that uniquely benefits. We hypothesized that patients with baseline HGF/cMet and EGFR pathway activation might disproportionately benefit from the combination of ficlatuzumab and cetuximab. However, here, as in other advanced cancer settings, plasma scMet was a marker of poor prognosis, while tumor cMet, HGF and cMet–HGF trended in the same direction, suggesting that these measures may be inadequate to predict benefit from dual pathway inhibition, or that this Phase I dose-finding study was underpowered to detect the impact of these prognostic markers. cMet is synthesized as a single-chain intracellular precursor and subsequently undergoes proteolytic processing during intracellular trafficking, leading to an α/β heterodimer at cell surface. The β chain in the extracellular domain can be proteolytically cleaved and released as scMet. Consistent with our findings, Gao et al. recently reported a significant correlation between scMet levels and NSCLC tumor cMet protein expression, and that patients with high scMet levels had poor OS [34]. In our study, tumor measures of HGF/cMet pathway activation were not associated with better survival outcomes. The lack of a significant correlation between cMet or HGF and the cMet–HGF complex suggests that there are multiple mechanisms involved in cMet activation, which may explain why patients with increased pathway activation did not uniquely respond to ficlatuzumab. While the VeriStrat proteomic classifier has mainly been used in the setting of NSCLC, a recent study showed prognostic value of VeriStrat for PFS and OS in afatinib (EGFR TKI)-treated recurrent/metastatic HNSCC patients, and OS in methotrexate-treated HNSCC patients. The VeriStrat proteomic classifier was not associated with either PFS or OS in our study [38]. Our biomarker results are consistent with circulating HGF as a robust pharmacodynamic marker of HGF mAb treatment [31,32,33,36,39], as we observed the expected increase in HGF after ficlatuzumab administration. The increase in HGF is thought to be due to stabilization of HGF upon forming a complex with ficlatuzumab.

The HGF/cMet signaling axis has pleiotropic effects on innate and adaptive immunity that are likely to be highly context dependent, and as such, we collected and cryopreserved PBMCs for exploratory immunophenotyping. Although the current study does not examine the specific effect of ficlatuzumab upon antitumor immunity, the immunophenotyping performed provides a potential immune profile both for patients who may be predisposed to and/or capable of sustaining a response to combined ficlatuzumab-cetuximab treatment. This beneficial immunologic state was characterized by the opposing presence of massive CD8^+^ T cell expansion seen in responders compared to the predominance of distinct myeloid cell subsets with low MHC class II expression, indicative of immunosuppressive activity, found at baseline in rapid progressors as well as upon ultimate progression in responders. A subset of cMet^+^ CD8^+^ T cells have been identified in murine tumor models and melanoma patients, where cMet expression is associated with increased cytolytic capacity and effector functions including IFNγ/TNFα and granzyme B/perforin production [40]. Administration of exogenous HGF in vitro, or in vivo exposure to HGF-producing tumors in mouse models inhibited effector functions of cMet^+^ CD8^+^ T cells [40]. Speculatively, cMet expression on CD8^+^ T cells may be associated with a specific developmental or functional state, such as antigen-experienced memory populations, and that subsequent HGF signaling may serve to inhibit ongoing inflammatory responses as a compensatory mechanism to subvert deleterious immunity during immune resolution [41]. Herein, ficlatuzumab may serve to potentiate cellular immunity by removing HGF-mediated immunosuppression in recurrent/metastatic HNSCC, allowing for expansion and potential activation of the T cell compartment. Concomitantly, HGF/cMet signaling conventionally enhances cellular motility and proliferation during tissue injury and/or organogenesis, and likely augments the wound healing functionality of myeloid cells. In the presence of malignant disease, such functionality serves to enhance angiogenesis, metastasis, and tumor cell survival. Tumor-associated macrophages and myeloid-derived suppressor cells are negatively associated with disease outcomes across numerous cancers [42,43]. In the current study, elevated myeloid cell composition prior to treatment may preclude potential beneficial effects of ficlatuzumab as inhibition of cMet–HGF may only serve to remove pro-tumorigenic stimuli but be insufficient to repolarize established immunosuppressive cells towards an inflammatory, tumoricidal phenotype.

## 4. Materials and Methods

### 4.1. Human Subjects Considerations

The protocol was approved by the University of Pittsburgh Institutional Review Board (PRO14100436) and registered with ClinicalTrials.gov (NCT02277197). All subjects provided written, informed consent. Primary inclusion criteria included recurrent/metastatic HNSCC from any primary site (except for Epstein–Barr virus-positive nasopharynx); known HPV status in the case of an oropharynx primary site as determined by p16 immunohistochemistry; measurable disease in accordance with the Response Evaluation Criteria in Solid Tumors (RECIST) version 1.1 [44]; age ≥ 18; Eastern Cooperative Oncology Group (ECOG) performance status 0–1; adequate end organ function and electrolytes; albumin ≥ 3 mg/dL; willingness to undergo baseline research biopsy. Clinical cetuximab resistance was required during cohort expansion, and was defined as either disease recurrence within 6 months of completing definitive cetuximab-radiation therapy or disease progression during or within 6 months of cetuximab in the recurrent/metastatic setting [45]. Cetuximab was not required to be the most recent systemic therapy received. Primary exclusion criteria included prior treatment with an HGF/cMet inhibitor; peripheral edema ≥ Grade 2; interstitial lung disease; any medical comorbidity that would interfere with the subject’s safety or compliance.

### 4.2. Study Design and Statistical Considerations

The primary objective of this Phase I study was to find the RP2D. The dose-finding study followed an adaptive, escalation–de-escalation, Narayana k-in-a-row design, with k set to 2 to target a 33% dose-limiting toxicity (DLT) rate [46]. This method was selected based on the superiority of its operating characteristics compared to other adaptive or rule-based designs (such as 3 + 3). Dose tiers are specified in Figure 1A. RP2D would be declared as the dose of ficlatuzumab that is close to but does not exceed a 33% DLT rate when administered with a standard, fixed dose of cetuximab. In the dose-finding phase, a total of either 8 or 14 patients would be treated. If no DLTs were observed among the first 8 patients (2 + 6 on Tiers 1 and 2), the upper 90% confidence bound for the estimated DLT rate at the highest dose tier would be 0.32 and less than the targeted toxicity rate of 0.33, thus Tier 2 would be declared the RP2D. If a DLT was observed at any time among the first 8 patients, then the escalation–de-escalation rules would be triggered and 14 DLT-evaluable subjects accrued. An intermediate tier (Tier 1.5) would be accrued only in the event that a DLT was observed and escalation–de-escalation required. After the enrollment of 14 patients to Tiers 1, 1.5, and 2, the RP2D would be estimated from DLTs across all dose levels by isotonic regression.

The observation period for identifying a DLT was the first cycle (4 weeks). A DLT was defined as any ≥ Grade 3 non-hematologic toxicity (except rash, infusion reaction, nausea, vomiting or diarrhea lasting < 48 hours, isolated AST or ALT elevation, or asymptomatic electrolyte abnormality); Grade 3 neutropenia with fever; Grade 3 thrombocytopenia with bleeding; Grade 4 neutropenia or thrombocytopenia; AST or ALT elevation ≥ 3-fold the upper limit of normal (ULN) with concurrent elevation of bilirubin ≥ 2-fold ULN; ficlatuzumab-related toxicity requiring a dose reduction or resulting in ≥ 2 missed doses.

According to the adaptive design, the total sample size required to establish the RP2D would range from 8 to 14. In order to conduct preliminary biomarker analyses, a minimum sample size of 12 biomarker and RECIST-evaluable patients was set. Preliminary oncologic efficacy was described as overall response rate (ORR) in accordance with RECIST v 1.1, progression-free survival (PFS), and OS in the total study population. PFS and OS were analyzed by the methods of Kaplan–Meier with 90% confidence intervals.

### 4.3. Treatment Plan

The protocol schema is presented in Figure 1A. One treatment cycle of ficlatuzumab and cetuximab was defined as four weeks. Subjects were dosed according to their assigned dose tier as specified. Cetuximab was dosed at 500 mg/m^2^ IV every 2 weeks, i.e., on Days 1 and 15 of a 28 day cycle. Subjects were assessed for response every 2 cycles, and continued treatment until disease progression. There was no intrapatient dose escalation. However, the dose of ficlatuzumab and/or cetuximab could be reduced for qualifying toxicities.

The dose and schedule for cetuximab was based on the established treatment paradigm in colorectal cancer, where clinical trials showed that biweekly dosing at 400–700 mg/m^2^ was well tolerated and that trough cetuximab levels for the 500 mg/m^2^ every 2 weeks, 600 mg/m^2^ every 2 weeks, and 250 mg/m^2^ weekly regimens were comparable [47,48]. In recurrent/metastatic HNSCC, a randomized Phase II study found that the biweekly dose of 500 mg/m^2^ resulted in similar efficacy to weekly dosing at 250 mg/m^2^, with no therapeutic advantage for 750 mg/m^2^ [49]. Due to convenience and comparable efficacy, clinical trials in HNSCC now incorporate biweekly dosing of cetuximab monotherapy at 500 mg/m^2^ (i.e., TPExtreme, NCT02268695) [50].

### 4.4. Biomarker Signaling Correlatives and Statistical Considerations

Blood samples were processed for isolation of sera, plasma and peripheral blood mononucleocytes (PBMCs). Individual enzyme-linked immunosorbent assays (ELISAs) for HGF (Quantikine ELISA kit, R&D Systems, Minneapolis, MN, USA, IL6 (Quantikine ELISA kit, R&D Systems) and scMet (ThermoScientific, Waltham, MA, USA) were used to quantify each analyte in duplicate using baseline plasma. All but two subjects had at least one additional blood sample collected post-treatment. Mean values were used for all analyses. VeriStrat testing was carried out on baseline serum samples at Biodesix, Inc. (Boulder, CO, USA), blinded to all clinical, treatment and outcome data. VeriStrat measures multiple circulating analytes by MALDI ToF mass spectrometry to assign VeriStrat Good or VeriStrat Poor classifications to blood samples which may predict clinical outcome of patients, independent of treatment and other prognostic factors [51].

To overcome the limitations of traditional immunohistochemistry (IHC) to evaluate activated cMet, we utilized the VeraTag^®^ proximity binding, dual antibody assay which measures the cMet–HGF receptor–ligand complex. VeraTag assays were also used to measure total cMet, HGF, EGFR and EGFR homodimers. All VeraTag assays were performed by Monogram Biosciences (South San Francisco, CA, USA) on formalin-fixed, paraffin-embedded (FFPE) tumor biopsies collected prior to study entry. Three blocks had insufficient tumor area for the assay. In one of these three cases, an archived biopsy was substituted prior to previous cetuximab exposure and was less likely to represent the cetuximab resistant state. FFPE blocks were sectioned at a thickness of 5 micron and placed on positively charged glass slides. Released fluorescent VeraTags were detected and quantified by capillary electrophoresis (CE). Following the VeraTag assay, the slides were hematoxylin and eosin (H&E) stained, tumor area identified and circled, and the fluorescent signal from the released VeraTag was normalized to sample buffer volume and tumor area to give units of relative fluorescence per square millimeter of tumor (RF/mm^2^). A panel of cell line controls was assayed together with the samples to control for batch-to-batch variability and allow for the comparison of samples over time. The following antibodies were used: cMet: anti-cMet rabbit mAb SP44 (Spring Bioscience, Pleasanton, CA, USA) and goat F(ab’)_2_ anti-rabbit IgG (Southern Biotech, Birmingham, AL, USA) labeled with a fluorescent VeraTag reporter via a disulfide bond; HGF: anti-HGF mouse mAb SBF5 (ThermoScientific), goat anti-HGF polyclonal IgG (R&D Systems) labeled with a fluorescent VeraTag reporter, and goat anti-mouse IgG (Southern Biotech) labeled with biotin; cMet–HGF complex: anti-cMET rabbit mAb SP44 (Spring Bioscience), goat anti-HGF polyclonal IgG (R&D Systems) labeled with a fluorescent VeraTag reporter, and goat F(ab’)_2_ anti-rabbit IgG (Southern Biotech) labeled with biotin; EGFR homodimer (H11D): equal concentrations of anti-HER1 rabbit mAb D38B1 (Cell Signaling Technology, Danvers, MA, USA) labeled with either a fluorescent VeraTag reporter or biotin. EGFR total (H1T), anti-EGFR rabbit mAb D38B1 (Cell Signaling Technology) and goat F(ab’)_2_ anti-mouse IgG (Southern Biotech) labeled with a fluorescent VeraTag reporter via a disulfide bond [52,53]. For both blood and tumor markers, levels below the limit of detection (LOD) were set to one-half the LOD for statistical analysis.

Five mechanistic biomarkers, including plasma (scMet, HGF) and tumor (cMet, HGF, and cMet–HGF complex) expression of an activated HGF/cMet pathway were pre-specified for specialized alpha spending and were considered significant if association with PFS or OS was ≤ 0.05 (unadjusted) in proportional hazards regression models. Additional exploratory biomarkers, including serum VeriStrat, circulating IL6, tumor EGFR expression and tumor EGFR homodimer expression were also evaluated as predictors of PFS or OS. Within-patient changes in tumor diameter were also tested for association of baseline tumor and blood markers by linear regression. The exploratory unplanned biomarker test *p* values were adjusted for false discovery by the method of Benjamini and Hochberg [54].

### 4.5. Immune Correlatives

Immunophenotyping was performed on cryopreserved PBMCs. Data collection was conducted on all samples at the same time to eliminate batch effects. All reagents were purchased from BioLegend unless specified. In total, 1–5×10^6^ cells per sample were stained in Cell-Staining Buffer using combinations of mAbs followed by labeling with amine-reactive viability dye (LiveDead, Molecular Probes, Eugene, OR). To determine leukocyte composition, cells were labeled extracellularly with mAbs specific for: CD3 (UCHT1; BD Biosciences, Franklin Lakes, NJ, USA), CD4 (RPA-T4), CD8a (RPA-T8), CD45RA (HI100), CD45RO (UCHL1), CD197 (G043H7), CD279 (eBioJ105; eBioscience, San Diego, CA, USA), HLA-DR (LN3; ThermoFisher, Waltham, MA, USA), CD11b (ICRF44), CD14 (61D3; ThermoFisher), CD15 (W6D3), CD16 (3G8), CD123 (6H6; BD), CD11c (B-ly6; BD), CD56 (5.1H11), CD19 (SJ25C1), CD25 (BC96), and CD127 (A019D5). Cells were then fixed, permeabilized, and labeled for intracellular expression of Ki-67 (SolA15; ThermoFisher) and FoxP3 (PCH101; ThermoFisher) using the True-Nuclear Transcription Factor Buffer Set (BioLegend, San Diego, CA, USA) per the manufacturer’s instructions. Data were collected on a four-laser Cytek Aurora spectral cytometer. FlowJo (BD Biosciences) software was used for conventionally gated data analysis. Lineage for DC identification consisted of CD3^+^, CD19^+^, and CD56^+^ cells. For computational analysis, samples were analyzed using Cytofkit package for R studio as previously described [55]. Briefly, viable, single cells were manually gated using FlowJo. Preprocessing was performed to generate expression matrix for each sample in a Flow Cytometry Standard (FCS) file. Parameters of interest were selected and FSC files were exported and uploaded into Cytofkit package. FCS files were transformed using automatic logicle transformation (autoLgcl) and merged in to one matrix using ceil. In total, 25,000 cells per sample were clustered using Rphenograph and visualized using t-Distributed Stochastic Neighbor Embedding (t-SNE). Samples were grouped per response to treatment (progressors vs. responders) and time point during therapy (baseline, response, progression), generating density plots within the collective t-SNE. Heatmaps were generated from Rphenograph using the expression heat map option depicting Rphenograph clusters and marker expression per cluster and per group.

## 5. Conclusions

In summary, the ficlatuzumab-cetuximab combination had an acceptable safety profile and showed promising antitumor activity in a refractory HNSCC patient population. Tumor-intrinsic biomarkers of HGF/cMet and EGFR pathway activation were not associated with survival outcomes in this therapeutic setting which was likely limited by the small sample size. Furthermore, the increase in peripheral CD8^+^ T cells associated with treatment response is the first report of potential immune modulatory activity of this combined regimen, warranting additional study. Our findings are limited by the small sample size of this Phase I study but support the ongoing randomized, Phase II study of ficlatuzumab with or without cetuximab in patients with cetuximab-resistant, recurrent or metastatic HSNCC (NCT03422536).

## Figures and Tables

**Table 1:** Baseline patient demographics and disease characteristics.

Patient Characteristics	*n* (%)
**Age** (Median, Range)	58.4 (46.7–80.1 years)
**Sex**	
Male	10 (77%)
Female	3 (23%)
**ECOG Performance Status**	
0	8 (62%)
1	5 (38%)
**Primary Tumor Site**	
Oral Cavity	1 (8%)
Oropharynx	4 (31%)
Hypopharynx	2 (15%)
Larynx	5 (38%)
External Auditory Canal	1 (8%)
**Platinum-Refractory**	
Yes	12 (92%)
No	1 (8%)
**Cetuximab-Resistant at Protocol Entry**	
Yes	12 (92%)
No	1 (8%)
**Tumor HPV Status**	
p16+ Oropharynx	1 (8%)
p16- Oropharynx and Non-Oropharynx	12 (92%)
**Previous Treatment with Anti-PD1/L1 mAb**	
Yes	5 (38%)
No	8 (62%)
**Baseline VeriStrat Status**	
Good	4 (33%)
Poor	9 (67%)

**Table 2:** Adverse events related to cetuximab plus ficlatuzumab by dose tier and grade in all patients that received at least one dose of drug (*n* = 3 at Tier 1; *n* = 10 at Tier 2).

Adverse Events	NCI CTCAE Grade			
	Grade 1–2 *n* (%)		Grade 3–4 *n* (%)	
	Tier 1	Tier 2	Tier 1	Tier2
**Constitutional**				
Flu-Like Symptoms	2 (67%)	3 (30%)	0	0
**Dermatologic**				
Acneiform Rash	3 (100%)	6 (60%)	0	0
**Hepatic**				
Hypoalbuminemia	0	5 (50%)	1 (33%)	0
**Infection**	1 (33%)	0	0	2 (20%)
**Metabolic**				
Hypomagnesemia	1 (33%)	3 (30%)	0	0
Hyponatremia	1 (33%)	4 (40%)	0	0
Hypophosphatemia	2 (67%)	2 (20%)	0	1 (10%)
**Vascular**				
Thromboembolism	0	0	1 (33%)	1 (10%)
Peripheral Edema	0	2 (20%)	0	1 (10%)
Head and Neck Lymphedema	1 (33%)	2 (20%)	0	0

**Table 3:** Hazards ratios and confidence intervals for proportional-hazards regression of PFS and OS for baseline circulating and tumor markers. HR= hazards ratio; CI= confidence intervals. Prespecified markers are indicated in bold. Adjusted *p* values are shown in parentheses for markers that were not prespecified in the protocol.

**Progression-Free Survival**			
**Covariate**	**HR**	**95% CI**	***p* value**
Circulating Biomarkers			
**scMet**	1.92	0.95–3.86	0.048
**HGF**	0.86	0.57–1.29	0.452
IL6	0.59	0.27–1.26	0.110 (0.337)
VeriStrat	1.54	0.41–5.81	0.517 (1.0)
Tumor Biomarkers			
**cMet**	2.09	0.70–6.24	0.172
**HGF**	1.65	0.37–7.33	0.508
**cMet–HGF**	2.11	0.54–8.14	0.273
H1T	2.80	1.03–7.56	0.023 (0.187)
H11D	1.14	0.86–1.51	0.335 (1.0)
**Overall Survival**			
**Covariate**	**HR**	**95% CI**	***p* value**
Circulating Biomarkers			
**scMet**	1.63	0.87–3.06	0.113
**HGF**	0.90	0.60–1.34	0.599
IL6	0.80	0.51–1.24	0.292 (0.790)
VeriStrat	0.89	0.25–3.06	0.852 (1.0)
Tumor Biomarkers			
**cMet**	1.58	0.51–4.92	0.422
**HGF**	1.47	0.32–6.61	0.618
**cMet–HGF**	1.84	0.45–7.56	0.390
H1T	1.94	0.80–4.71	0.13 (0.790)
H11D	1.13	0.85–1.50	0.39 (0.790)

## Supplementary Materials

The following are available online at https://www.mdpi.com/2072-6694/12/6/1537/s1, Figure S1: Mean plasma HGF levels (ng/mL) per patient and according to treatment cycle as measured by ELISA.

## References

[1] Bray F., Ferlay J., Laversanne M., Brewster D.H., Mbalawa C.G., Kohler B., Piñeros M., Steliarova-Foucher E., Swaminathan R., Antoni S. (2015). Cancer Incidence in Five Continents: Inclusion criteria, highlights from Volume X and the global status of cancer registration. Int. J. Cancer.

[2] Vermorken J.B., Mesia R., Rivera F., Remenár É., Kawecki A., Rottey S., Erfan J., Zabolotnyy D., Kienzer H.-R., Cupissol D. (2008). Platinum-Based Chemotherapy plus Cetuximab in Head and Neck Cancer. N. Engl. J. Med..

[3] Ferris R.L., Blumenschein G., Fayette J., Guigay J., Colevas A.D., Licitra L., Harrington K., Kasper S., Vokes E.E., Even C. (2016). Nivolumab for Recurrent Squamous-Cell Carcinoma of the Head and Neck. N. Engl. J. Med..

[4] Cohen E.E.W., Soulières D., le Tourneau C., Dinis J., Licitra L., Ahn M.-J., Soria A., Machiels J.-P., Mach N., Mehra R. (2019). Pembrolizumab versus methotrexate, docetaxel, or cetuximab for recurrent or metastatic head-and-neck squamous cell carcinoma (KEYNOTE-040): A randomised, open-label, phase 3 study. Lancet.

[5] Grandis J.R., Melhem M.F., Gooding W.E., Holst V.A., Wagener M.M., Drenning S.D., Day R., Tweardy D.J. (1998). Levels of TGF-α and EGFR Protein in Head and Neck Squamous Cell Carcinoma and Patient Survival. J. Natl. Cancer Inst..

[6] Chung C.H., Ely K., McGavran L., Varella-Garcia M., Parker J., Parker N., Jarrett C., Carter J., Murphy B.A., Netterville J. (2006). Increased Epidermal Growth Factor Receptor Gene Copy Number Is Associated With Poor Prognosis in Head and Neck Squamous Cell Carcinomas. J. Clin. Oncol..

[7] Vermorken J.B., Herbst R.S., León X., Amellal N., Baselga J. (2008). Overview of the efficacy of cetuximab in recurrent and/or metastatic squamous cell carcinoma of the head and neck in patients who previously failed platinum-based therapies. Cancer.

[8] Allegra C.J., Jessup J.M., Somerfield M.R., Hamilton S.R., Hammond E.H., Hayes D.F., McAllister P.K., Morton R.F., Schilsky R.L. (2009). American Society of Clinical Oncology Provisional Clinical Opinion: Testing for KRAS Gene Mutations in Patients With Metastatic Colorectal Carcinoma to Predict Response to Anti–Epidermal Growth Factor Receptor Monoclonal Antibody Therapy. J. Clin. Oncol..

[9] Licitra L., Störkel S., Kerr K.M., van Cutsem E., Pirker R., Hirsch F.R., Vermorken J., von Heydebreck A., Esser R., Celik I. (2013). Predictive value of epidermal growth factor receptor expression for first-line chemotherapy plus cetuximab in patients with head and neck and colorectal cancer: Analysis of data from the EXTREME and CRYSTAL studies. Eur. J. Cancer.

[10] Licitra L., Mesia R., Rivera F., Remenár É., Hitt R., Erfán J., Rottey S., Kawecki A., Zabolotnyy D., Benasso M. (2011). Evaluation of EGFR gene copy number as a predictive biomarker for the efficacy of cetuximab in combination with chemotherapy in the first-line treatment of recurrent and/or metastatic squamous cell carcinoma of the head and neck: EXTREME study. Ann. Oncol..

[11] Peruzzi B., Bottaro D.P. (2006). Targeting the c-Met Signaling Pathway in Cancer. Clin. Cancer Res..

[12] Knowles L.M., Stabile L.P., Egloff A.M., Rothstein M.E., Thomas S.M., Gubish C.T., Lerner E.C., Seethala R.R., Suzuki S., Quesnelle K.M. (2009). HGF and c-Met participate in paracrine tumorigenic pathways in head and neck squamous cell cancer. Clin. Cancer Res..

[13] Mandal M., Myers J.N., Lippman S.M., Johnson F.M., Williams M.D., Rayala S.K., Ohshiro K., Rosenthal D.I., Weber R.S., Gallick G.E. (2008). Epithelial to mesenchymal transition in head and neck squamous carcinoma. Cancer.

[14] Basu D., Nguyen T.-T.K., Montone K.T., Zhang G., Wang L.-P., Diehl J.A., Rustgi A.K., Lee J.T., Weinstein G.S., Herlyn M. (2010). Evidence for mesenchymal-like sub-populations within squamous cell carcinomas possessing chemoresistance and phenotypic plasticity. Oncogene.

[15] Xu H., Stabile L.P., Gubish C.T., Gooding W.E., Grandis J.R., Siegfried J.M. (2011). Dual blockade of EGFR and c-Met abrogates redundant signaling and proliferation in head and neck carcinoma cells. Clin. Cancer Res..

[16] Seiwert T.Y., Jagadeeswaran R., Faoro L., Janamanchi V., Nallasura V., El Dinali M., Yala S., Kanteti R., Cohen E.E., Lingen M.W. (2009). The MET receptor tyrosine kinase is a potential novel therapeutic target for head and neck squamous cell carcinoma. Cancer Res..

[17] Wilson T.R., Fridlyand J., Yan Y., Penuel E., Burton L., Chan E., Peng J., Lin E., Wang Y., Sosman J. (2012). Widespread potential for growth-factor-driven resistance to anticancer kinase inhibitors. Nature.

[18] Engelman J.A., Zejnullahu K., Mitsudomi T., Song Y., Hyland C., Park J.O., Lindeman N., Gale C.-M., Zhao X., Christensen J. (2007). MET Amplification Leads to Gefitinib Resistance in Lung Cancer by Activating ERBB3 Signaling. Science.

[19] Yano S., Yamada T., Takeuchi S., Tachibana K., Minami Y., Yatabe Y., Mitsudomi T., Tanaka H., Kimura T., Kudoh S. (2011). Hepatocyte Growth Factor Expression in EGFR Mutant Lung Cancer with Intrinsic and Acquired Resistance to Tyrosine Kinase Inhibitors in a Japanese Cohort. J. Thorac. Oncol..

[20] Takahashi N., Yamada Y., Furuta K., Honma Y., Iwasa S., Takashima A., Kato K., Hamaguchi T., Shimada Y. (2014). Serum levels of hepatocyte growth factor and epiregulin are associated with the prognosis on anti-EGFR antibody treatment in KRAS wild-type metastatic colorectal cancer. Br. J. Cancer.

[21] Yamada T., Takeuchi S., Kita K., Bando H., Nakamura T., Matsumoto K., Yano S. (2012). Hepatocyte Growth Factor Induces Resistance to Anti-Epidermal Growth Factor Receptor Antibody in Lung Cancer. J. Thorac. Oncol..

[22] Tanaka H., Kimura T., Kudoh S., Mitsuoka S., Watanabe T., Suzumura T., Tachibana K., Noguchi M., Yano S., Hirata K. (2011). Reaction of plasma hepatocyte growth factor levels in non-small cell lung cancer patients treated with EGFR-TKIs. Int. J. Cancer.

[23] Trotta A.M., Ottaiano A., Romano C., Nasti G., Nappi A., de Divitiis C., Napolitano M., Zanotta S., Casaretti R., D’Alterio C. (2016). Prospective Evaluation of Cetuximab-Mediated Antibody-Dependent Cell Cytotoxicity in Metastatic Colorectal Cancer Patients Predicts Treatment Efficacy. Cancer Immunol. Res..

[24] López-Albaitero A., Lee S.C., Morgan S., Grandis J.R., Gooding W.E., Ferrone S., Ferris R.L. (2009). Role of polymorphic Fc gamma receptor IIIa and EGFR expression level in cetuximab mediated, NK cell dependent in vitro cytotoxicity of head and neck squamous cell carcinoma cells. Cancer Immunol. Immunother..

[25] Faden D.L., Concha-Benavente F., Chakka A.B., McMichael E.L., Chandran U., Ferris R.L. (2019). Immunogenomic correlates of response to cetuximab monotherapy in head and neck squamous cell carcinoma. Head Neck.

[26] Papaccio F., Della-Corte C.M., Viscardi G., di Liello R., Esposito G., Sparano F., Ciardiello F., Morgillo F. (2018). HGF/MET and the Immune System: Relevance for Cancer Immunotherapy. Int. J. Mol. Sci..

[27] Okunishi K., Dohi M., Nakagome K., Tanaka R., Mizuno S., Matsumoto K., Miyazaki J.-I., Nakamura T., Yamamoto K. (2005). A novel role of hepatocyte growth factor as an immune regulator through suppressing dendritic cell function. J. Immunol..

[28] Benkhoucha M., Santiago-Raber M.-L., Schneiter G., Chofflon M., Funakoshi H., Nakamura T., Lalive P.H. (2010). Hepatocyte growth factor inhibits CNS autoimmunity by inducing tolerogenic dendritic cells and CD25+Foxp3+ regulatory T cells. Proc. Natl. Acad. Sci. USA.

[29] Bauman J.E., Arias-Pulido H., Lee S.-J., Fekrazad M.H., Ozawa H., Fertig E.J., Howard J., Bishop J., Wang H., Olson G.T. (2013). A phase II study of temsirolimus and erlotinib in patients with recurrent and/or metastatic, platinum-refractory head and neck squamous cell carcinoma. Oral Oncol..

[30] To Y., Dohi M., Matsumoto K., Tanaka R., Sato A., Nakagome K., Nakamura T., Yamamoto K. (2002). A Two-way Interaction between Hepatocyte Growth Factor and Interleukin-6 in Tissue Invasion of Lung Cancer Cell Line. Am. J. Respir. Cell Mol. Boil..

[31] Tarhini A.A., Rafique I., Floros T., Tran P., Gooding W.E., Villaruz L.C., Burns T.F., Friedland D.M., Petro D.P., Farooqui M. (2017). Phase 1/2 study of rilotumumab (AMG 102), a hepatocyte growth factor inhibitor, and erlotinib in patients with advanced non-small cell lung cancer. Cancers.

[32] Zhang Y., Doshi S., Zhu M. (2015). Pharmacokinetics and pharmacodynamics of rilotumumab: A decade of experience in preclinical and clinical cancer research. Br. J. Clin. Pharmacol..

[33] Tan E.H., Lim W.-T., Ahn M.-J., Ng Q.-S., Ahn J.S., Tan D.S.-W., Sun J.-M., Han M., Payumo F.C., McKee K. (2018). Phase 1b Trial of Ficlatuzumab, a Humanized Hepatocyte Growth Factor Inhibitory Monoclonal Antibody, in Combination With Gefitinib in Asian Patients With NSCLC. Clin. Pharmacol. Drug Dev..

[34] Gao H.-F., Li A.-N., Yang J.-J., Chen Z.-H., Xie Z., Zhang X.-C., Su J., Lou N.-N., Yan H.-H., Han J.-F. (2017). Soluble c-Met Levels Correlated With Tissue c-Met Protein Expression in Patients With Advanced Non–Small-Cell Lung Cancer. Clin. Lung Cancer.

[35] Mok T.S., Geater S.L., Su W.-C., Tan E.-H., Yang J.C.-H., Chang G.-C., Han M., Komarnitsky P., Payumo F., Garrus J.E. (2016). A Randomized Phase 2 Study Comparing the Combination of Ficlatuzumab and Gefitinib with Gefitinib Alone in Asian Patients with Advanced Stage Pulmonary Adenocarcinoma. J. Thorac. Oncol..

[36] Patnaik A., Weiss G.J., Papadopoulos K.P., Hofmeister C.C., Tibes R., Tolcher A., Isaacs R., Jac J., Han M., Payumo F.C. (2014). Phase I ficlatuzumab monotherapy or with erlotinib for refractory advanced solid tumours and multiple myeloma. Br. J. Cancer.

[37] Wang Q., Yang S., Wang K., Sun S.-Y. (2019). MET inhibitors for targeted therapy of EGFR TKI-resistant lung cancer. J. Hematol. Oncol..

[38] Cohen E.E.W., Licitra L.F., Burtness B., Fayette J., Gauler T., Clement P.M., Grau J.J., Del Campo J.M., Mailliez A., Haddad R.I. (2017). Biomarkers predict enhanced clinical outcomes with afatinib versus methotrexate in patients with second-line recurrent and/or metastatic head and neck cancer. Ann. Oncol..

[39] Tabernero J., Elez M.E., Herranz M., Rico I., Prudkin L., Andreu J., Mateos J., Carreras M.J., Han M., Gifford J. (2014). A Pharmacodynamic/Pharmacokinetic Study of Ficlatuzumab in Patients with Advanced Solid Tumors and Liver Metastases. Clin. Cancer Res..

[40] Benkhoucha M., Molnarfi N., Kaya G., Belnoue E., Bjarnadóttir K., Dietrich P., Walker P.R., Martinvalet D., Derouazi M., Lalive P.H. (2017). Identification of a novel population of highly cytotoxic c-Met-expressing CD8 + T lymphocytes. EMBO Rep..

[41] Sugimoto M.A., Vago J., Perretti M., Teixeira M.M. (2019). Mediators of the Resolution of the Inflammatory Response. Trends Immunol..

[42] Williams C.B., Yeh E.S., Soloff A.C. (2016). Tumor-associated macrophages: Unwitting accomplices in breast cancer malignancy. NPJ Breast Cancer.

[43] Yen B.L.-J., Yen M.-L., Hsu P.-J., Liu K.-J., Wang C.-J., Bai C.-H., Sytwu H.-K. (2013). Multipotent Human Mesenchymal Stromal Cells Mediate Expansion of Myeloid-Derived Suppressor Cells via Hepatocyte Growth Factor/c-Met and STAT3. Stem Cell Rep..

[44] Eisenhauer E.A., Therasse P., Bogaerts J., Schwartz L., Sargent D., Ford R., Dancey J., Arbuck S., Gwyther S., Mooney M. (2009). New response evaluation criteria in solid tumours: Revised RECIST guideline (version 1.1). Eur. J. Cancer.

[45] Stabile L., Egloff A., Gibson M., Gooding W., Ohr J., Zhou P., Rothenberger N., Wang L., Geiger J., Flaherty J. (2017). IL6 is associated with response to dasatinib and cetuximab: Phase II clinical trial with mechanistic correlatives in cetuximab-resistant head and neck cancer. Oral Oncol..

[46] Ivanova A., Montazer-Haghighi A., Mohanty S.G., Durham S.D. (2002). Improved up-and-down designs for phase I trials. Stat. Med..

[47] Tabernero J., Ciardiello F., Rivera F., Rodriguez-Braun E., Ramos F.J., Martinelli E., Vega-Villegas M.E., Roselló S., Liebscher S., Kisker O. (2010). Cetuximab administered once every second week to patients with metastatic colorectal cancer: A two-part pharmacokinetic/pharmacodynamic phase I dose-escalation study. Ann. Oncol..

[48] Tabernero J., Pfeiffer P., Cervantes A. (2008). Administration of Cetuximab Every 2 Weeks in the Treatment of Metastatic Colorectal Cancer: An Effective, More Convenient Alternative to Weekly Administration?. Oncologist.

[49] Staff J., Sherman E., Lisa D., Agarwal N., Algazy K., Brockstein B., Langer C., Lim D., Mehra R., Rajan S.K. (2012). A randomized phase II study of cetuximab every 2 weeks at either 500 or 750 mg/m2 for patients with recurrent or metastatic head and neck squamous cell cancer. J. Natl. Compr. Cancer Netw..

[50] Guigay J., Fayette J., Mesia R., Lafond C., Saada-Bouzid E., Geoffrois L., Martin L., Cupissol D., Capitain O., Castanie H. (2019). TPExtreme randomized trial: TPEx versus Extreme regimen in 1st line recurrent/metastatic head and neck squamous cell carcinoma (R/M HNSCC). J. Clin. Oncol..

[51] Carbone D.P., Ding K., Roder H., Grigorieva J., Roder J., Tsao M.-S., Seymour L., Shepherd F.A. (2012). Prognostic and Predictive Role of the VeriStrat^®^ Plasma Test in Patients with Advanced Non-Small Cell Lung Cancer Treated with Erlotinib or Placebo in the NCIC Clinical Trials Group BR.21 Trial. J. Thorac. Oncol..

[52] Dua R., Zhang J., Parry G., Penuel E. (2011). Detection of Hepatocyte Growth Factor (HGF) Ligand-c-MET Receptor Activation in Formalin-Fixed Paraffin Embedded Specimens by a Novel Proximity Assay. PLoS ONE.

[53] Shi Y., Huang W., Tan Y., Jin X., Dua R., Penuel E., Mukherjee A., Sperinde J., Pannu H., Chenna A. (2009). A Novel Proximity Assay for the Detection of Proteins and Protein Complexes: Quantitation of HER1 and HER2 Total Protein Expression and Homodimerization in Formalin-fixed, Paraffin-Embedded Cell Lines and Breast Cancer Tissue. Diagn. Mol. Pathol..

[54] Benjamini Y., Hochberg Y. (1995). Controlling the False Discovery Rate: A Practical and Powerful Approach to Multiple Testing. J. R. Stat. Soc. Ser. B.

[55] Chen H., Lau M.C., Wong M.T., Newell E.W., Poidinger M., Chen J. (2016). Cytofkit: A Bioconductor Package for an Integrated Mass Cytometry Data Analysis Pipeline. PLoS Comput. Boil..

