# Supplementary material for: Phase I Study of Ficlatuzumab and Cetuximab in Cetuximab-Resistant, Recurrent/Metastatic Head and Neck Cancer
Source: Cancers (Basel). 2020 Jun 11;12(6):1537. doi: 10.3390/cancers12061537 (PMC7352434; doi:10.3390/cancers12061537)
Supplement: Supplementary file 1 [file cancers-12-01537-s001.pdf]

Supplementary Materials

Phase I Study of Ficlatuzumab and Cetuximab in Cetuximab-Resistant, Recurrent/Metastatic Head and Neck Cancer

Julie E. Bauman, James Ohr, William E. Gooding, Robert L. Ferris, Umamaheswar Duvvuri, Seungwon Kim, Jonas T. Johnson, Adam C. Soloff, Gerald Wallweber, John Winslow, Autumn Gaither-Davis, Jennifer R. Grandis and Laura P. Stabile

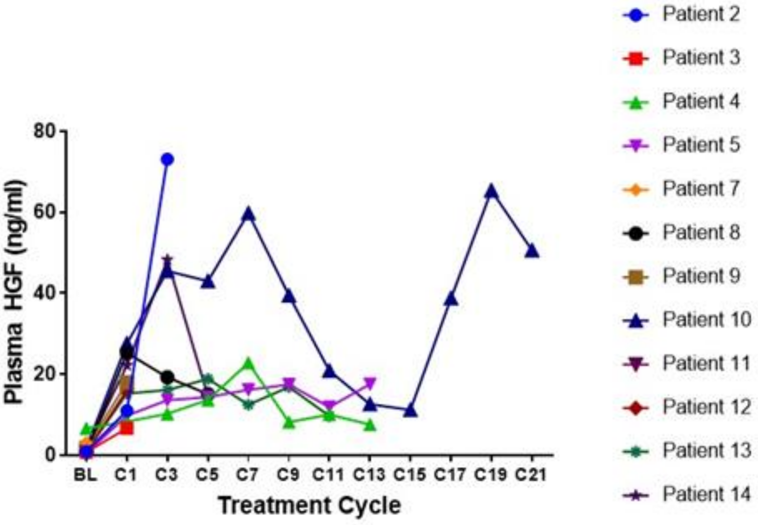

Figure S1. Mean plasma HGF levels (ng/mL) per patient and according to treatment cycle as measured by ELISA.

© 2020 by the authors. Licensee MDPI, Basel, Switzerland. This article is an open access article distributed under the terms and conditions of the Creative Commons Attribution (CC BY) license (http://creativecommons.org/licenses/by/4.0/).
